# Role of RONS and eIFs in Cancer Progression

**DOI:** 10.1155/2021/5522054

**Published:** 2021-07-05

**Authors:** Yasmeen Ahmed Salaheldin, Salma Sayed Mohamed Mahmoud, Ebenezeri Erasto Ngowi, Vivian Aku Gbordzor, Tao Li, Dong-Dong Wu, Xin-Ying Ji

**Affiliations:** ^1^Department of Pathology, Faculty of Medicine, Ain Shams University, Cairo 11517, Egypt; ^2^International Joint Laboratory for Nucleoprotein Gene Regulation, School of Basic Medical Sciences, Henan University, Kaifeng, Henan 475004, China; ^3^Department of Biochemistry, Faculty of Medicine, Ain Shams University, Cairo 11517, Egypt; ^4^Department of Biological Sciences, Faculty of Science, Dar es Salaam University College of Education, Dar es Salaam 2329, Tanzania; ^5^Kaifeng Municipal Key Laboratory of Cell Signal Transduction, Engineering Centre for Tumor Molecular Medicine, Henan University, Kaifeng, Henan 475004, China; ^6^School of Stomatology, Henan University, Kaifeng, Henan 475004, China; ^7^Kaifeng Key Laboratory of Infection and Biological Safety, School of Basic Medical Sciences, Henan University, Kaifeng, Henan 475004, China

## Abstract

Various research works have piled up conflicting evidence questioning the effect of oxidative stress in cancer. Reactive oxygen and nitrogen species (RONS) are the reactive radicals and nonradical derivatives of oxygen and nitrogen. RONS can act as a double-edged weapon. On the one hand, RONS can promote cancer initiation through activating certain signal transduction pathways that direct proliferation, survival, and stress resistance. On the other hand, they can mitigate cancer progression via their resultant oxidative stress that causes many cancer cells to die, as some recent studies have proposed that high RONS levels can limit the survival of cancer cells during certain phases of cancer development. Similarly, eukaryotic translation initiation factors are key players in the process of cellular transformation and tumorigenesis. Dysregulation of such translation initiation factors in the form of overexpression, downregulation, or phosphorylation is associated with cancer cell's altering capability of survival, metastasis, and angiogenesis. Nonetheless, eIFs can affect tumor age-related features. Data shows that alternating the eukaryotic translation initiation apparatus can impact many downstream cellular signaling pathways that directly affect cancer development. Hence, researchers have been conducting various experiments towards a new trajectory to find novel therapeutic molecular targets to improve the efficacy of anticancer drugs as well as reduce their side effects, with a special focus on oxidative stress and initiation of translation to harness their effect in cancer development. An increasing body of scientific evidence recently links oxidative stress and translation initiation factors to cancer-related signaling pathways. Therefore, in this review, we present and summarize the recent findings in this field linking certain signaling pathways related to tumorigeneses such as MAPK and PI3K, with either RONS or eIFs.

## 1. Introduction

Cancer is considered the second leading cause of mortality worldwide according to the World Health Organization. Chemotherapy and radiotherapy can help in the management of some types of cancer, but the net outcome of oncological diseases is still far from satisfactory, which directs most of the contemporary medical researchers to focus on this field. Several studies have been conducted to find new molecular therapeutic strategies, to improve the efficacy of cancer treatment and reduce the side effects. Recent research has been focusing on oxidative stress and initiation of translation as a potential target in cancer treatment. Some types of cancer such as acute lymphocytic leukemia and neuroblastoma are more common in young adults [1, 2], with around 50% of testicular cancer cases occurring in men between the age of 20 and 34 [3]. Cancer diseases are more common among the elderly population due to longer exposure to various risk factors such as exposure to chemicals, radiation, chronic inflammation, unhealthy lifestyle, accumulation of altered macromolecules, and decreased immunity [4]. Patients younger than 20 years old account for only 1.4% of all newly diagnosed cancer cases according to Global Cancer Observatory.

Cancer cells alter mitochondrial dynamics, which include mitochondrial fission and fusion that primarily determine the balance between mitochondrial energy production and cell death programs. Cancer cells cause mitochondrial dynamics to resist apoptosis and adjust their bioenergetic and biosynthetic requirements to support tumor initiation and transformation properties such as autophagy, proliferation, migration, and therapeutic resistance. Microenvironmental stresses impact intratumoral heterogeneity and impose stem-like traits on cancer cells. Major cancer-related pathways such as mitogen-activated protein kinase and phosphatidylinositol-3-kinase, which are both activated by reactive oxygen and nitrogen species, can reprogram mitochondrial function and dynamics. Cancer cells rely on such reprogramming for sustained proliferation, the capacity to metastasize and to resist apoptosis, thus positioning mitochondria as a pivot for major cancer traits [5].

Aerobic eukaryotes are faced with a phenomenon known as the oxygen paradox, where they cannot survive without oxygen, but at the same time, oxygen is considered lethal to their survival. This is due to the presence of unpaired electrons. The mitochondrial electron transport chain produces water from the reduction of oxygen. However, the univalent reduction of oxygen produces reactive intermediates which are frequently encountered within the physiological cellular state [6]. Reactive oxygen species are those reactive intermediates, in other words, partially reduced oxygen molecules that can give rise to functional and morphological cellular disturbances being capable of reacting with almost every component of the cell [7]. On the one hand, RONS can mutate nucleic acid and damage cellular components that raised the assumption many years ago stating that both cellular aging and cancer initiation may reflect accumulated damage of RONS over periods [8]. On the other hand, Gonskikh and Polacek demonstrated that RONS can be beneficial. They explained that reduced protein synthesis caused by oxidative stress is associated with increased production of specific proteins that improve the overall cellular performance [9].

**Figure 1:**
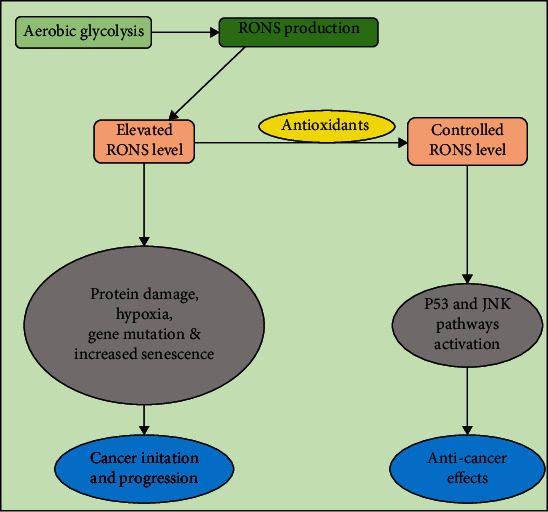
A schematic presentation of RONS levels in the cell and their impact, the reasonable amounts of RONS are a key player for activating protective signaling pathways whereas elevated RONS is considered lethal to most cellular functions and may lead to cancer development. RONS: reactive oxygen and nitrogen species; P53: tumor suppressor; JNK: Jun N-terminal kinase.

RONS are the reactive radicals and nonradical derivatives of oxygen and nitrogen. They are produced in all aerobic cells and play a key role in cancer. They both originate from endogenous and exogenous sources. Exogeneous sources include air and water pollution, drugs (e.g., cyclosporins, tacrolimus, gentamycin, and bleomycin), tobacco, alcohol, heavy metals, industrial solvents, cooking (e.g., smoked meat, waste oil, and fat), and radiation, which are metabolized inside the body into free radicals, whereas endogenous sources consist of nicotinamide adenine dinucleotide phosphate oxidase, myeloperoxidase, lipoxygenase, and angiotensin II [10, 11]. Oxidative stress is a result of antioxidants and RONS imbalance due to either depletion of antioxidants or accumulation of RONS as shown in (Figure 1). RONS such as superoxide, peroxyl radical, hydrogen peroxide, hydroxyl radical, and peroxy nitrite can react with nucleic acids, proteins, and lipids, thus resulting in cell and tissue damage.

Different subcellular compartments including both enzymatic and nonenzymatic reactions produce RONS. The enzymatic reactions include superoxide dismutase, glutathione peroxidase, guaiacol peroxidase, peroxiredoxins, and enzymes of the ascorbate-glutathione cycle, such as ascorbate peroxidase, monodehydroascorbate reductase, dehydroascorbate reductase, and glutathione reductase, whereas nonenzymatic examples include vitamin C, vitamin E, and glutathione molecule [12]. Two main cellular organelles, namely, the endoplasmic reticulum and the mitochondria, are intimately involved in RONS production and their metabolism. They both constitute a fundamental role in redox regulation [13]. Cellular enzymes known as NADPH oxidases produce a considerable amount of RONS in humans [6]. Other cellular sources of RONS include neutrophils, monocytes, cardiomyocytes, endothelial cells, xanthine oxidases, cytochrome P450, lipoxygenases, and nitric oxide synthases [14, 15]. Peroxisomes also produce RONS via both beta-oxidation of fatty acids and flavin oxidase activity [16]. RONS are involved in various physiological processes and essential protective mechanisms that living organisms use for their survival. The protective mechanisms obviously would be the role of the immune defense [17] and vascular tone [18] which aims at maintaining a state of homeostasis. Living organisms strive to keep those highly reactive molecules under tight control with the help of a complex system of antioxidants [19]. Accumulated evidence over time suggested that RONS has a pivotal role in the determination of cell fate, acting as second messengers and modifying various signaling molecules.

Oxidative stress refers to the incapability of the cell to detoxify free radicals produced, resulting in inefficient cellular performance. The mechanism used by the cell in response to oxidant effects is to restore the balance by promoting or inhibiting genes encoding defensive enzymes, transcription factors, and structural proteins [20]. The accumulation of these reactive species affects normal cellular pathways and therefore plays a positive role in cancer by damaging the amino acids, DNA, and lipids that act as building blocks of the body. Intracellular RONS are important components of intracellular signaling cascades [21]. A recent study suggested that RONS act as a double agent by promoting cancer initiation through activating signaling pathways that control proliferation, survival, and stress resistance and on the other side by suppressing cancer initiation and progression via oxidative stress that kills many cancer cells [22].

Initiation of translation is the complex and rate-determining step of protein synthesis. This process is conserved in all eukaryotes, and it involves multiple eukaryotic initiation factors, including but not limited to eIF1, eIF1a, eIF2, eIF2b, eIF3, eIF4a, eIF4e, eIF4g, eIF4b, eIF4h, eIF5, eIF5b, and eIF6 [23]. eIFs are proteins, most of which are composed of several subunits, and have a major role in the regulation of the translation initiation machinery [24]. Major events in initiation comprise (1) formation of a 43S preinitiation complex which consists of a 40S ribosomal subunit and binds to eIFs 1, 1A, 3, and 5 and also a ternary complex consisting of the initiating methionyl-tRNA which binds to eIF2-Guanosine triphosphate (eIF2-GTP-MettRNAi); (2) assembling of elF4F complex (eIF4E, eIF4G, eIF4A) on mRNA 5′ m7GpppN cap; (3) eIF4F complex facilitating the recruitment of the 43S PIC to the mRNA via eIF4G–eIF3 interaction to form the 43S mRNA initiation complex; (4) in the 43S mRNA initiation complex, scanning of the mRNA 5′ of the untranslated region in the 5′ to 3′ direction to the initiation codon; (5) initiation codon recognition and 48S complex formation; (6) eIF5B promoting the hydrolysis of eIF2-bound guanosine triphosphate, the displacement of eIFs, and the joining of a 60S subunit; (7) GTP hydrolysis by eIF5B and release of eIF1A and guanosine diphosphate-bound eIF5B from assembled elongation-competent 80S ribosomes; (8) formation of an active 80S ribosome to initiate protein synthesis; and (9) eIF5A promoting peptide bond formation and translation elongation. The inactive eIF2-GDP is recycled to active eIF2-GTP by GTP recycling factor eIF2B [23–27]. Dysregulation of translation initiation factors in the form of overexpression, downregulation, or phosphorylation affects cancer cell survival, metastasis, and tumor angiogenesis and aging-related features [23, 27].

Signaling pathways and translation initiation factors have represented a promising aspect for further studies in cancer treatment, as their dysregulation promotes cancer progression. In addition, in vivo trials have provided up-and-coming results, including some that have already moved to the final phase of clinical trial. RONS play a double agent role, depending on their cumulative amount within the cell. The impact of elevated amounts of RONS is of greater importance when it comes to developing new cancer therapeutics; however, targeting RONS requires determining the threshold level of lethal RONS in different cells, opening an opportunity for more research to be done concerning the mechanisms and relevant applications of the proposed approaches.

Blocking dysregulated signaling pathways such as (PI3K/Ak strain transforming/mechanistic target of rapamycin) pathway and translation regulators by kinase inhibitors have yielded promising outcomes as cancer treatment targets. Also, researchers aiming at targeting dysregulated eIFs as a cancer therapy focus on eIF4 complex. This may be achieved in many ways such as suppressing eIF4E activity or targeting its subunits [23]. It may help in controlling the disease and discovering a new vision for cancer treatment in combination with conventional chemotherapeutics. Future studies should focus on determining the clear mechanism and the role of initiation factors in aging. Another study demonstrated that overexpression of forkhead box O6 inhibits the migration and progression of breast cancer cells [28]. Understanding crosstalk between the signaling pathways is the major challenge in targeting signaling pathways, and also, the adverse events associated with drugs make treatment more complicated.

## 2. RONS in Cancer

At low levels, RONS can be beneficial to cells activating signaling pathways that promote survival. In contrast, at higher levels, RONS can damage or even kill cells by oxidizing cellular components including proteins, lipids, and most importantly nucleic acids. However, recent studies have proposed that high RONS levels can also limit the survival of cancer cells during certain phases of cancer initiation and progression [22]. In some cancer cells, high RONS levels can be attributed to hypoxia, sustained mitochondrial respiration, unfolded protein response, and oncogenesis [29]. Classically, RONS have been demonstrated to promote various types of cancers. This was attributed to their ability to induce DNA damage and thus enhancing the rate of tumor-causing mutations and genetic instability besides their proinflammatory effect. Recent evidence suggests that cancer cells are more sensitive than normal cells to elevated RONS levels [30] and that they rely on glutathione and thioredoxin for protection [31]. In some cancers, including melanomas, oxidative stress acted as a barrier to distant metastasis [32].

**Figure 2:**
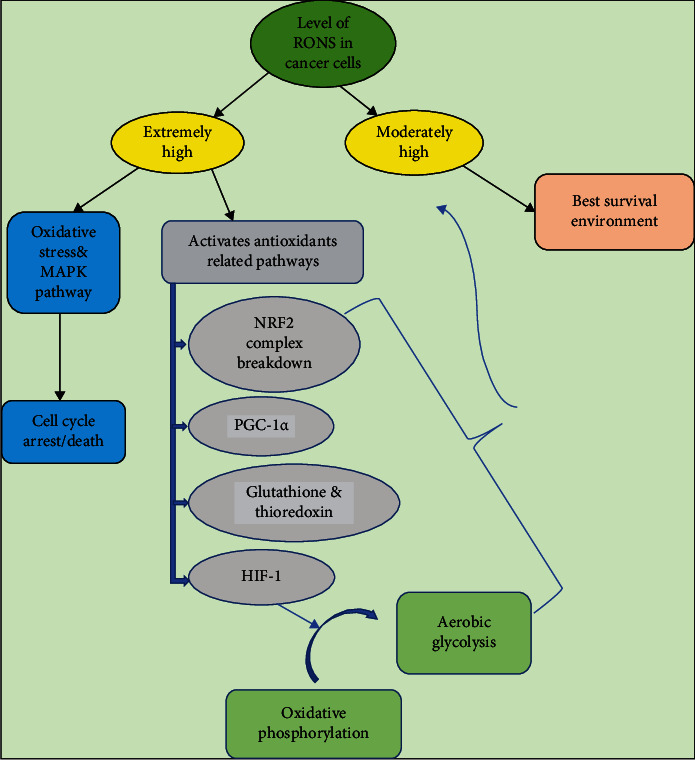
A schematic illustration of RONS regulation within cancer cells depicting some of the mechanisms used to reduce the extreme accumulation of RONS to reach the optimum level for cellular performance and survival. Excessive accumulation of RONS enhances cell death notably via ASK1/JNK/P38 MAPK pathway activation. RONS: reactive oxygen and nitrogen species; NRF2: nuclear factor erythroid-derived 2-like 2; PGC-1*α*: peroxisome proliferator-activated receptor gamma coactivator one alpha; HIF-1: hypoxic inducible factor one.

Besides, the survival of tumor cells outside of a normal tissue context requires adaptation to the metabolism of different microenvironments. Cancer cells depend on a variety of mechanisms to suppress RONS and to cope with oxidative stress [22]. Cancer cell uses several mechanisms to avoid the extreme accumulation of radicals as shown in Figure 2. For example, hypoxia-inducible factor, which is a transcription factor that responds to the decrease in available cellular oxygen in response to elevated RONS levels, has been shown to mediate the shift of oxidative phosphorylation of anaerobic glycolysis aiming to decrease RONS levels and eventually increase the survival of cancer cells during their metastasis to the lungs [33]. In a new study, peroxisome proliferator-activated receptor gamma coactivator 1-alpha protein, a key molecule activated by RONS production, involved in mitochondrial biogenesis and antioxidant enzymes activation was identified to promote chemoresistance in response to RONS generated by exposure of cells to ovarian sphere-forming culture conditions [34]. Moreover, nuclear factor-like, an inducible antioxidant program, is a redox stress-sensitive transcription factor that induces several antioxidant and detoxification genes. The activation of the NRf2 antioxidant program in response to cellular stressors results in a decrease in RONS levels. DeNicola and colleagues have demonstrated that several endogenous oncogenes such as KRAS, BRAF, and Myc in mice can actively induce the NRf2 expression, promoting a RONS detoxification program and hence creating a more “reduced” intracellular environment. This program is what the authors have suggested is required for tumor initiation [35]. NRF2-deficient cancer cells showed impaired cancer progression by globally suppressing protein translation due to unopposed oxidative stress [36]. Multiple transcription factors such as activation transcription factor 4 also cooperate to induce an antioxidant response that promotes survival. NRF2 and ATF4 promote the expression of serine/glycine biosynthesis enzymes to increase glutathione synthesis, which reduces oxidative stress and promotes survival during metastasis [37]. Tumors from approximately 15% of patients with lung cancer harbor somatic mutations in Kelch-like ECH-associated protein 1 that prevent effective NRF2 repression [38].

Some other tumor suppressors also act partly by suppressing RONS production. BCR-ABL-transformed cells (cells with gene translocation between chromosomes 9 and 22, also known as Philadelphia chromosome) show increased intracellular RONS, as well as oxidative DNA damage and chromosomal fragmentation [39]. The oxidative inhibition of phosphatase and TENsin homolog, PTEN, a tumor suppressor gene, by abnormally elevated levels of RONS in many tumors could functionally impair the tumor-suppressing activity of the enzyme, enhancing tumor development [40]. Another study demonstrated a cysteine residue involving the mechanism by which Maspin, another tumor suppressor, reduces RONS production, and RONS scavenging was associated with the inhibition of extracellular signal-regulated kinase 1/2 [41].

### 2.1. Nuclear Factor Kappa-Light-Chain Enhancer of Activated B Pathway

NF-*κ*B is a transcription factor that is considered crucial in many processes including inflammatory response, cellular adhesion, differentiation, proliferation, autophagy, senescence, and apoptosis. The disorder of NF-*κ*B has already been confirmed to be associated with cancer [42]. The bidirectional interrelation was found between both RONS and NF-*κ*B. The NF-*κ*B pathway may be activated by at least two distinct pathways named canonical and noncanonical pathways. RONS affect NF-*κ*B in multiple manners, e.g., RONS can activate the noncanonical pathway, leading to NF-*κ*B activation [43]. At the same time, the canonical pathway can be inactivated, leading to NF-*κ*B inhibition. NF-*κ*B can influence the RONS levels by increasing the expression of various antioxidant proteins [44]. A recent study found that the NF-*κ*B pathway can also be activated by a tumor necrosis factor receptor that is regulated by eIF3b in human osteosarcoma cells [45].

### 2.2. Mitogen-Activated Protein Kinase (MAPK) Pathway

Another signaling pathway altered by RONS is the MAPK pathway. MAPK cascades are major intracellular signal transduction pathways that play an important role in various cellular processes such as cell growth, differentiation, development, cell cycle, survival, and cell death. The MAPK/ERK pathway is activated mainly by growth factors, and this depends primarily on RAS phosphorylation [46]. RONS have been shown to activate the receptors of epidermal growth factor and platelet-derived growth factor in the absence of their corresponding ligands, which can stimulate RAS and the subsequent activation of the MAPK pathway [47]. Also, it has been demonstrated that RONS generated by commensal bacteria in the activated DUSP3 gene by oxidation on Cys-124 results in MAPK pathway activation. DUSP3 gene maps to a region that contains the BRCA-1 locus, which confers susceptibility to breast and ovarian cancer [48]. RONS was also found to activate c-JUN N-terminal kinase pathway that is one of the MAPK cascades [49, 50].

### 2.3. PI3K/AKT Pathway

The PI3K/AKT pathway is involved in many critical cellular functions, including protein synthesis, cell cycle progression, proliferation, apoptosis, autophagy, and drug resistance [51]. RONS do not only activate PI3K directly but also concurrently inactivates PTEN, which inhibits the activation of AKT. PTEN is a tumor suppressor gene on chromosome 10, and its mutation is linked to many cancers; RONS can also enhance PTEN to enter the proteolytic degradation pathway [52].

### 2.4. Calcium Signaling Pathway

In eukaryotic cells, calcium acts as one of the most versatile signals involved in the control of cellular processes and functions, such as contraction, secretion, metabolism, gene expression, cell survival, and cell death. A bidirectional interrelation was found between both calcium and RONS [53]. The primary role of calcium is to promote adenosine triphosphate synthesis and RONS generation in mitochondria via stimulating the Krebs cycle enzymes and oxidative phosphorylation [54]. Calcium ion regulates several extramitochondrial RONS generating enzymes, such as NADPH oxidase and nitric oxide synthase [55]. Besides, calcium modulates RONS clearance processes by regulating the antioxidant defense system. Calcium ions can directly activate antioxidant enzymes such as catalase and glutathione reductase, increase the level of superoxide dismutase, and induce mitochondrial glutathione release. Meanwhile, calmodulin, a ubiquitous calcium-binding protein, activates catalase in the presence of calcium and downregulates hydrogen peroxide levels [56]. Moreover, RONS can also influence calcium signaling by oxidizing cysteine thiol groups of the calcium channel [57].

Mitochondria Permeability Transition Pore, a large, nonspecific channel spanning the inner and outer mitochondrial membranes, is known to control the lethal permeability changes that initiate mitochondrial-driven death [58]. RONS modulate mPTP opening in two ways: firstly, by directly oxidizing different sites in its structure [55] and secondly by either indirectly increasing the mitochondrial calcium concentration [59] or by activating the Jun N-terminal kinase pathway [50]. In a recent study, opening mPTP was shown to cause RONS to increase and promote apoptosis in cancer cells [60].

### 2.5. Protein Kinases

RONS function in various cellular processes via oxidizing the sulfhydryl groups of cysteine residues in various protein kinases such as protein kinase C/D, calmodulin-dependent protein kinase II, and receptor tyrosine kinases such as insulin receptor, epidermal growth factor receptor, and platelet growth factor receptor, resulting in their activation [61, 62]. RONS play a dual role in both stimulation and inactivation of PKC following its concentration: higher doses of oxidants react with catalytically important cysteine residues inactivating PKC whereas low doses induce stimulation of PKC activity [63].

### 2.6. Ubiquitination/Proteasome System

Ubiquitination/proteasome system plays an indispensable role in a variety of biological processes such as regulation of the cell cycle, inflammatory responses, immune response, protein misfolding, and endoplasmic reticulum-associated degradation of proteins [64]. Oxidative stress affects the process of ubiquitination in different ways. First, the rapid depletion of reduced glutathione and improvement of the levels of oxidized glutathione upon exposure to oxidative stress result in the oxidation of cysteine residues at the active sites of the ubiquitin-activating enzymes E1 and E2 and the generation of mixed disulfide bonds, which block their binding to ubiquitin thus altering its function [65]. Second, it has also been reported that bacteria elicit RONS generation in epithelial cells that inactivate the Ubc12 enzyme by preventing the neddylation of cullin-1, rendering it unable to carry out ubiquitination and thus making it inactive [66]. Third, the proteasome itself is considered a target of oxidative stress, and it was proposed that the 26S proteasome is more susceptible than the 20S proteasome to oxidative inactivation [67].

Meanwhile, ubiquitination impacted by RONS has been studied in some cancers. 3-Hydroxy butyrate dehydrogenase 2 is considered to be an important tumor suppressor in gastric cancer. BDH2 was found to regulate the level of intracellular RONS to mediate the PI3K/Akt pathway through Keap1/Nrf2 signaling, thereby inhibiting the growth of gastric cancer. Mechanistically, BDH2 promoted Keap1 interaction with Nrf2 to increase the ubiquitination of Nrf2 consequently increasing the level of RONS, thereby inhibiting the phosphorylation of AKT and mTOR [68]. Another study handled ubiquitination in thyroid cancer, and they found that vitamin C kills thyroid cancer cells by inhibiting MAPK/ERK and PI3K/AKT pathways via a RONS-dependent mechanism. They suggested that vitamin C eradicated BRAF wild-type thyroid cancer cells through a ROS-mediated decrease in the activity of epidermal growth factor/epidermal growth factor receptor-MAPK/ERK signaling and an increase in AKT ubiquitination [69]. Sajadimajd and Khazaei studied the ubiquitination of NRF2 and its correlation with RONS. They found that under normal conditions, NRF2 is commonly degraded in the cytoplasm by interaction with Keap1 inhibitor as an adaptor for ubiquitination factors. However, a high amount of RONS activates tyrosine kinases to dissociate NRF2: Keap1 complex, nuclear import of NRF2, and coordinated activation of cytoprotective gene expression [70].

### 2.7. FOXO Apoptotic Pathway

Cancer cells are known by their ability to escape cancer drugs by hijacking autophagy; in the light of recent studies, it is idealized that blocking autophagy can help in increasing apoptosis through transcriptional factor FOXO3a which links the two processes, by maintaining autophagy equilibrium and controlling a gene responsible for making an apoptosis-facilitating protein called p53 upregulated modulator of apoptosis [71].

FOXO transcription factors are involved in inducing apoptotic injury and RONS regulation in cancer cells. Overexpression of Survivin, an antiapoptotic protein acting on the FOXO3 apoptotic pathway, has been reported in neuroblastoma cells. FOXO3a is shown to prevent reactive oxygen species accumulation and shift energy production from oxidative phosphorylation to glycolysis [72]. The pathway is activated by cellular oxidative stress via PI3K signaling. An elevated level of superoxide dismutase 2 is associated with increased FOXO activity [73], while NAD-dependent deacetylase sirtuin-3 was demonstrated to promote the FOXO3a expression by inhibiting the wingless-related integration site/*β*-catenin pathway. They suggested that upregulation of FOXO6 was shown to inhibit epithelial-mesenchymal transition and migration of breast cancer cells and vice versa [28].

### 2.8. Pentose Phosphate Pathway

The PPP, which branches from glycolysis at the first committed step of glucose metabolism, is required for the synthesis of ribonucleotides and is a major source of NADPH. PPP is crucial for cancer cell survival and lipid biosynthesis. Given that NADPH is central to oxidative stress resistance, cancer cells modulate the PPP to maintain their anabolic demands and keep a state of redox homeostasis. Recently, several neoplastic lesions were shown to have evolved to facilitate the flux of glucose into the pentose phosphate pathway [74]. Circulating microRNA-21, a homotetrameric protein that was found to be highly expressed in many solid tumors, catalyzes the oxidative decarboxylation of malate to yield carbon dioxide and pyruvate, with concomitant reduction of NAD^+^ or NADP^+^. In non-small-cell lung cancer cell lines, MIR-21 depletion caused an inhibition of cell proliferation with induction of cell death accompanied by increased RONS. Furthermore, MIR-21 knockdown impacts phosphoinositide-dependent kinase-1 and PTEN expression, leading to PI3k pathway inhibition [75].

The big picture reflecting the contribution of various mediators plus local environmental factors seems to be the actual determinant for RONS-induced consequences in both physiology and pathology; therefore, it is essential to unravel the not-yet-well-understood parts of this intricate picture for a better understanding of the RONS induced alterations [19]. The net effect of RONS on cancer reflects a complex combination of adaptive and maladaptive consequences within the cells and their environment [22].

## 3. eIFs in Cancer

Messenger RNA translation or protein synthesis plays a major role in the regulation of the eukaryotic gene expression [76]. Many studies confirmed that dysregulation of the translational machinery, especially in the initiation, can lead to abnormal gene expression and uncontrolled cell growth resulting in cancer [25, 77–79]. Regulation of the translational process is mainly achieved by the rate-limiting initiation step, which is organized by multiple eukaryotic initiation factors [20]. Out of many eIFs, only six are involved in translation initiation.  Table 1 describes the six factors involved and the role of each one.

Alteration in initiation rate can occur by a change in initiation factors' availability or activation of oncogenic signaling pathways, such as PI3K/AKT/mTOR and MAPK pathways [80]. Many relations have been found between the translation machinery and some oncogenes such as Myc and RAS families and tumor suppressors such as PTEN and p53 [77–79].

Translation initiation factors also have a major role in cellular transformation and tumorigenesis. Dysregulation of translation initiation factors in the form of overexpression, downregulation, or phosphorylation is involved in cancer cell survival, metastasis, and tumor angiogenesis [23, 27]. The regulation of initiation factors including overexpression of eIF4A, eIF4E, and eIF4G; downregulation of eIF4E-binding protein levels; and phosphorylation of eIF2 is involved in various types of cancer as shown in Table 2 [23, 27, 78, 81]. And still, the specific role played by increased initiation factors, levels, or activity in cancer behavior remains poorly understood.

### 3.1. MAPK/MAPK-Interacting Kinases 1-2 Pathway

MNK1 and MNK2 phosphorylate eIF4E on a single residue Ser209. MAPK and ERK pathways activate MNKs in response to stress and mitogens, respectively [82]. Hyperphosphorylation of eIF4E can lead to an increase in specific mRNA translation that encodes prosurvival proteins such as myeloid cell leukemia-1, invasion, and epithelial to mesenchymal transition promoting proteins and cytokines [83]. Experimental results revealed that complete loss of eIF4E phosphorylation in the absence of MNK1 and MNK2 in the mouse model may delay the development of tumorigenesis [84]. Hence, the eIF4F complex has an important role in tumorigenesis, which is affected by many oncogenic pathways [85].

### 3.2. PI3K/AKT Pathway

E74-like factor 4 complex is a multisubunit which consists of eIF4E, eIF4A, and eIF4G. It is considered as a rate-limiting component in the initiation of translation as its role is to recruit small ribosomal subunits and related factors (43S, PICs) to the 5′ end of mRNA [23]. The mTOR has a major role in the regulation of eIF4E [86]. As hyperphosphorylation of eIF4E binding proteins by mTOR enables its dissociation from eIF4E, so eIF4F can interact with eIF4G and form ELF4 complex and continue the translation process [85].

In cancer, activation of oncogenes (e.g., AKT) or loss of tumor suppressors (e.g., PTEN) leads to activation of mTORC1, a hallmark of a cancer cell, which enhances cell proliferation, survival, and invasion [86]. Hyperactivated mTOR can lead to overexpression of eIF4E, which promotes the translation of specific mRNAs which are involved in angiogenesis, cell proliferation, and cell survival, namely, vascular endothelial growth factor-A, cMyc, and B-cell lymphoma 2, respectively [87]. Cellular stresses, such as amino acid deprivation and hypoxia, which are common in tumors, downregulate mTORC1 activity, preventing the formation of the eIF4F complex, and thus downregulating protein synthesis [85].

Previous studies have confirmed that the overexpression of eIF4F or loss of eIF4E-binding protein 1 is the key feature of most poor prognostic and drug-resistant cancer cells [88, 89]. Some studies suggested that increased mTOR activity can also lead to overexpression of eIF4A1 [23, 25]. On the other hand, eIF6 is necessary for ribosome biogenesis in the nucleus. Several studies have described eIF6 as an important factor in age-related diseases such as colorectal cancer, malignant pleural mesothelioma, and breast cancer. In CRC and MPM, eIF6 was overexpressed compared to nonneoplastic tissues, suggesting a key contribution to carcinogenesis. In a recent study, the knockdown of eIF6 in adenocarcinoma and squamous cell carcinoma led to pre-rRNA processing and ribosomal 60S maturation defects, and in non-small-cell lung cancer, there was upregulation of eIF6 [90].

### 3.3. Miscellaneous Pathways Involving eIFs in Cancer

Other studies confirmed that silencing eIF3a reverses the malignant phenotype of human lung and breast cancer cell lines and downregulates the cyclin dependent kinase inhibitor p27 [91]. Silencing eIF3d also showed a role in limiting the proliferation and invasion of cancer cells by suppressing Wnt/B-catenin signaling and cyclin dependent kinase-1 [92, 93]. The overexpression of eIF3f may lead to suppression of AKT and ERK signaling, an increase of p53 protein levels, and inhibition of clusters in protein expression, which also promotes cancer cell proliferation and reduces chemosensitivity [94]. Recent studies also suggest that prolonged eIF2*α* phosphorylation, which prevents the conversion of GDP to GTP by increasing the affinity of eIF2B for eIF2-GDP, results in blocking of protein synthesis [27]. eIF2*α* can promote cell survival, transformation, and drug resistance, whereas other studies suggest that eIF2*α* phosphorylation can trigger apoptosis [95, 96]. Moreover, eIF6 is upregulated and active in many human cancers and may be regulated by protein kinase C by phosphorylation on S235 [97].

## 4. RONS Impacting eIFs

RONS can affect eIFs in different ways. A study confirmed that specific translational control driven by eIF6 is essential for adjusting a set of RONS-controlling genes in megakaryocytes. Specifically, genes coding for the mitochondrial electron transport chain complex I and complex IV and those involved in RONS production. They identified the pathways of the mitochondrial electron transport chain and oxidative phosphorylation as the most significantly impaired [98]. Another group of researchers investigated the mechanism linking eIF5A2 and RONS. They found that the inhibition of eIF5A2 affects epithelial mesenchymal transition progression so that it decreases the invasion and metastasis of hepatocellular carcinoma cells via RONS-related pathways [99]. Moreover, it is suggested that radiation-induced autophagy is a prosurvival response initiated by oxidative stress and mediated by eIF2A kinase 3 [100].

## 5. Conclusion

Oxidative stress and initiation of translation fundamentally influence oncogenesis and age-related diseases by altering the relevant downstream pathways and that can be used to our privilege in treating oncologic diseases. Oxidative stress has been described as one of the main causes of cellular damage and mutations resulting in decreased cellular performance; however, these radicals existing in reasonable amounts within the cell can play a crucial role in the activation of various signaling pathways. Initiation of translation is an important step in synthesizing a protein, altering of which can affect the quantity and quality of the proteins produced. To maintain normal cellular functioning, these proteins need to be degraded; failure of that degradation will lead to accumulated proteins, consequently increasing the possibility of cancer. Moreover, dysregulation of translation initiation factors has been associated with cancer progression; however, more studies are needed to determine the involved mechanism. Hence, future studies should focus more on harnessing the initiation of translation and reactive radicals to target cancer cells. Targeting the pathways associated with the initiation of translation and RONS should be given the utmost priority.

## Figures and Tables

**Table 1:** Functions of different eIFs.

Protein	Function	Refs.
eIF1	mRNA screening and delivery of tRNA.	[23, 101]

eIF2	Initiation codon recognition.	[27]
eIF2b	Allows for the next initiation to occur (returns the released GDP to GTP).	

eIF3	Recruiting translation factors and 40S ribosome subunits to the mRNA.	[23, 24, 102]

eIF4F	Multisubunit complex:	[24, 103]
(i) eIF4E	CAP binding activity, a rate-limiting factor.	
(ii) eIF4G	Scaffolding protein and interaction partner for other factors.	
(iii) eIF4A	RNA helicase.	
(iv) eIF4B and eIF4H	mRNA secondary structure unwinding.	

eLF5	Translation elongation and bond formation.	[23]

eIF6	Prevents 60S subunit association with 40S subunit in the absence of mRNA (antiassociation factor).	[25]

eIF: eukaryotic initiation factor; mRNA: messenger RNA; tRNA: tranfer RNA; GDP: glutamine dipeptide; GTP: glutamine tripeptide; CAP: catabolite activator protein.

**Table 2:** Dysregulated eIFs in different types of cancer.

Protein	Form of dysregulation	Resultant cancer	Refs.
eIF1	Overexpression	HCC	[104]
	Mutation	Thyroid cancer	[105]

eIF2 alpha	Increased Phosphorylation	Oropharyngeal	[95]
	Overexpression	Gastrointestinal	[106]
		NSCL	[107]
		Lymphoma	[23]
		Brain tumor	[108]
		Thyroid carcinoma	[109]

eIF3A	Overexpression	Colorectal	[110]

eIF3B		Esophageal	[111]

eIF3C		Glioma	[112]

eIF3D		Breast	[92]
		Prostate	[93]
		Gastric	[113]

eIF3H		HCC	[114]

eIF3I		Head and neck	[115]

eIF3M		Colorectal	[116]

eIF3E	Downregulation	Breast	[23]

eIF3F		Pancreatic	[117]

eIF4E	Overexpression	Brain	[108]
		Endometrial	[118]
		Head and neck	[119]
		Bladder	[120]
		Cervical	[121]
		Prostate	[122]
		Colon	[123]
		Liver	[124]
		Lymphoma	[125]
		Esophagus	[126]
		Gastric	[127]

eIF4G		Nasopharyngeal	[128]
		Breast	[80, 129]
		Squamous cell lung cancer	[130]
		Cervical cancer	[81]
		Melanoma	[131]

eIF4A	Downregulation	Breast	[132]
		Lung	[133]

eIF4B	Overexpression	B-cell lymphoma	[134]

eIF4H		Lung	[135]

eIF5	Overexpression	HCC	[136]
		Glioblastoma	[137]
		Lung	[138]
		Urinary bladder	[139]
		Ovarian	[140]
		Colorectal	[141]

eIF6	Overexpression	Leukemia	[142]
		Ovarian serous	[143]

HCC: hepatocellular carcinoma; NSCL: non-small-cell lung cancer.

## Data Availability

Data are deposited in a repository.

## References

[1] McNeer J. L., Bleyer A. (2018). Acute lymphoblastic leukemia and lymphoblastic lymphoma in adolescents and young adults. *Pediatric Blood & Cancer*.

[2] Olecki E., Grant C. N. (2019). MIBG in neuroblastoma diagnosis and treatment. *Seminars in Pediatric Surgery*.

[3] Chieffi P. (2019). An up-date on novel molecular targets in testicular germ cell tumors subtypes. *Intractable & rare diseases research*.

[4] Liguori I., Russo G., Curcio F. (2018). Oxidative stress, aging, and diseases. *Clinical Interventions in Aging*.

[5] Senft D., Ze’ev A. R. (2016). Regulators of mitochondrial dynamics in cancer. *Current Opinion in Cell Biology*.

[6] Lassègue B., San Martín A., Griendling K. K. (2012). Biochemistry, physiology, and pathophysiology of NADPH oxidases in the cardiovascular system. *Circulation Research*.

[7] Martinez-Cayuela M. (1995). Oxygen free radicals and human disease. *Biochimie*.

[8] Harman D. (1956). Aging: a theory based on free radical and radiation chemistry. *Journal of Gerontology*.

[9] Gonskikh Y., Polacek N. (2017). Alterations of the translation apparatus during aging and stress response. *Mechanisms of Ageing and Development*.

[10] Genestra M. (2007). Oxyl radicals, redox-sensitive signalling cascades and antioxidants. *Cellular Signalling*.

[11] Phaniendra A., Jestadi D. B., Periyasamy L. (2015). Free radicals: properties, sources, targets, and their implication in various diseases. *Indian Journanl of Clinical Biochemistry*.

[12] McCord J. M., Fridovich I. (1969). Superoxide Dismutase. *The Journal of Biological Chemistry*.

[13] Hernández-García D., Wood C. D., Castro-Obregón S., Covarrubias L. (2010). Reactive oxygen species: a radical role in development?. *Free Radical Biology & Medicine*.

[14] Cubero F. J., Nieto N. (2012). Arachidonic acid stimulates TNF*α* production in Kupffer cells via a reactive oxygen species-pERK1/2-Egr1-dependent mechanism. *American Journal of Physiology-Gastrointestinal and Liver Physiology*.

[15] Azzam E. I., Jay-Gerin J. P., Pain D. (2012). Ionizing radiation-induced metabolic oxidative stress and prolonged cell injury. *Cancer Letters*.

[16] Schrader M., Fahimi H. D. (2006). Peroxisomes and oxidative stress. *Biochimica et Biophysica Acta*.

[17] Dröge W. (2002). Free radicals in the physiological control of cell function. *Physiological Reviews*.

[18] Liu Y., Zhao H., Li H., Kalyanaraman B., Nicolosi A. C., Gutterman D. D. (2003). Mitochondrial sources of H2O2 generation play a key role in flow-mediated dilation in human coronary resistance arteries. *Circulation Research*.

[19] Alfadda A. A., Sallam R. M. (2012). Reactive oxygen species in health and disease. *Journal of Biomedicine and Biotechnology*.

[20] Birben E., Sahiner U. M., Sackesen C., Erzurum S., Kalayci O. (2012). Oxidative stress and antioxidant defense. *World Allergy Organization Journal*.

[21] Bratic A., Larsson N. G. (2013). The role of mitochondria in aging. *The Journal of Clinical Investigation*.

[22] Gill J. G., Piskounova E., Morrison S. J. (2016). Cancer, oxidative stress, and metastasis. *Cold Spring Harbor Symposia on Quantitative Biology*.

[23] Ali M. U., Ur Rahman M. S., Jia Z., Jiang C. (2017). Eukaryotic translation initiation factors and cancer. *Tumor Biology*.

[24] Spilka R., Ernst C., Mehta A. K., Haybaeck J. (2013). Eukaryotic translation initiation factors in cancer development and progression. *Cancer Letters*.

[25] Chu J., Cargnello M., Topisirovic I., Pelletier J. (2016). Translation initiation factors: reprogramming protein synthesis in cancer. *Trends in Cell Biology*.

[26] Jackson R. J., Hellen C. U., Pestova T. V. (2010). The mechanism of eukaryotic translation initiation and principles of its regulation. *Nature Reviews Molecular Cell Biology*.

[27] de la Parra C., Walters B. A., Geter P., Schneider R. J. (2018). Translation initiation factors and their relevance in cancer. *Current Opinion in Genetics & Development*.

[28] Li R., Quan Y., Xia W. (2018). SIRT3 inhibits prostate cancer metastasis through regulation of FOXO3A by suppressing Wnt/*β*-catenin pathway. *Experimental Cell Research*.

[29] Gorrini C., Harris I. S., Mak T. W. (2013). Modulation of oxidative stress as an anticancer strategy. *Nature Reviews Drug Discovery*.

[30] Raj L., Ide T., Gurkar A. U. (2011). Selective killing of cancer cells by a small molecule targeting the stress response to ROS. *Nature*.

[31] Harris I. S., Brugge J. S. (2015). The enemy of my enemy is my friend. *Nature*.

[32] Piskounova E., Agathocleous M., Murphy M. M. (2015). Oxidative stress inhibits distant metastasis by human melanoma cells. *Nature*.

[33] Zhao T., Zhu Y., Morinibu A. (2014). HIF-1-mediated metabolic reprogramming reduces ROS levels and facilitates the metastatic colonization of cancers in lungs. *Scientific Reports*.

[34] Kim B., Jung J. W., Jung J. (2017). PGC1*α* induced by reactive oxygen species contributes to chemoresistance of ovarian cancer cells. *Oncotarget*.

[35] DeNicola G. M., Karreth F. A., Humpton T. J. (2011). Oncogene-induced Nrf2 transcription promotes ROS detoxification and tumorigenesis. *Nature*.

[36] Chio I. I. C., Jafarnejad S. M., Ponz-Sarvise M. (2016). NRF2 promotes tumor maintenance by modulating mRNA translation in pancreatic cancer. *Cell*.

[37] DeNicola G. M., Chen P.-H., Mullarky E. (2015). NRF2 regulates serine biosynthesis in non–small cell lung cancer. *Nature Genetics*.

[38] Hayes J. D., McMahon M. (2009). NRF2 and KEAP1 mutations: permanent activation of an adaptive response in cancer. *Trends in Biochemical Sciences*.

[39] Nowicki M. O., Falinski R., Koptyra M. (2004). BCR/ABL oncogenic kinase promotes unfaithful repair of the reactive oxygen species–dependent DNA double-strand breaks. *Blood*.

[40] Benhar M., Engelberg D., Levitzki A. (2002). ROS, stress-activated kinases and stress signaling in cancer. *EMBO Reports*.

[41] Mahajan N., Shi H. Y., Lukas T. J., Zhang M. (2013). Tumor-suppressive maspin functions as a reactive oxygen species scavenger: importance of cysteine residues. *Journal of Biological Chemistry*.

[42] Baldwin A. S. (2012). Regulation of cell death and autophagy by IKK and NF-*κ*B: critical mechanisms in immune function and cancer. *Immunological Reviews*.

[43] Kim J.-H., Na H.-J., Kim C.-K. (2008). The non-provitamin A carotenoid, lutein, inhibits NF-*κ*B-dependent gene expression through redox-based regulation of the phosphatidylinositol 3-kinase/PTEN/Akt and NF-*κ*B-inducing kinase pathways: role of H2O2 in NF-*κ*B activation. *Free Radical Biology and Medicine*.

[44] Reynaert N. L., van der Vliet A., Guala A. S. (2006). Dynamic redox control of NF-kappaB through glutaredoxin-regulated S-glutathionylation of inhibitory kappaB kinase beta. *Proceedings of the National Academy of Sciences*.

[45] Choi Y. J., Lee Y. S., Lee H. W., Shim D. M., Seo S. W. (2017). Silencing of translation initiation factor eIF3b promotes apoptosis in osteosarcoma cells. *Bone & joint research*.

[46] Plotnikov A., Zehorai E., Procaccia S., Seger R. (2011). The MAPK cascades: signaling components, nuclear roles and mechanisms of nuclear translocation. *Biochimica et Biophysica Acta (BBA) - Molecular Cell Research*.

[47] Lei H., Kazlauskas A. (2009). Growth factors outside of the platelet-derived growth factor (PDGF) family employ reactive oxygen species/Src family kinases to activate PDGF receptor *α* and thereby promote proliferation and survival of cells. *Journal of Biological Chemistry*.

[48] Wentworth C. C., Alam A., Jones R. M., Nusrat A., Neish A. S. (2011). Enteric commensal bacteria induce extracellular signal-regulated kinase pathway signaling via formyl peptide receptor-dependent redox modulation of dual specific phosphatase 3. *Journal of Biological Chemistry*.

[49] Davies C., Tournier C. (2012). Exploring the function of the JNK (c-Jun N-terminal kinase) signalling pathway in physiological and pathological processes to design novel therapeutic strategies. *Biochemical Society Transactions*.

[50] Castro-Caldas M., Carvalho A. N., Rodrigues E., Henderson C., Wolf C. R., Gama M. J. (2012). Glutathione S-transferase pi mediates MPTP-induced c-Jun N-terminal kinase activation in the nigrostriatal pathway. *Molecular Neurobiology*.

[51] Qiu X., Cheng J. C., Chang H. M., Leung P. C. (2014). COX2 and PGE2 mediate EGF-induced E-cadherin-independent human ovarian cancer cell invasion. *Endocrine-Related Cancer*.

[52] Leslie N. R., Downes C. P. (2002). PTEN: the down side of PI 3-kinase signalling. *Cellular Signalling*.

[53] Naranjo J. R., Mellström B. (2012). Ca^2+^-dependent transcriptional control of Ca^2+^ homeostasis. *Journal of Biological Chemistry*.

[54] Lewis A., Hayashi T., Su T. P., Betenbaugh M. J. (2014). Bcl-2 family in inter-organelle modulation of calcium signaling; roles in bioenergetics and cell survival. *Journal of Bioenergetics and Biomembranes*.

[55] Zhang J., Wang X., Vikash V. (2016). ROS and ROS-Mediated Cellular Signaling. *Oxidative Medicine and Cellular Longevity*.

[56] Gordeeva A. V., Zvyagilskaya R. A., Labas Y. A. (2003). Cross-talk between reactive oxygen species and calcium in living cells. *Biochemistry (Moscow)*.

[57] Chaube R., Hess D. T., Wang Y. J. (2014). Regulation of the skeletal muscle ryanodine receptor/Ca2+-release channel RyR1 by S-palmitoylation. *Journal of Biological Chemistry*.

[58] Morciano G., Giorgi C., Bonora M. (2015). Molecular identity of the mitochondrial permeability transition pore and its role in ischemia-reperfusion injury. *Journal of Molecular and Cellular Cardiology*.

[59] Voronina S., Okeke E., Parker T., Tepikin A. (2014). How to win ATP and influence Ca^2+^ signaling. *Cell Calcium*.

[60] NavaneethaKrishnan S., Rosales J. L., Lee K. Y. (2018). Loss of Cdk5 in breast cancer cells promotes ROS-mediated cell death through dysregulation of the mitochondrial permeability transition pore. *Oncogene*.

[61] Eisenberg-Lerner A., Kimchi A. (2012). PKD is a kinase of Vps34 that mediates ROS-induced autophagy downstream of DAPk. *Cell Death & Differentiation*.

[62] Sag C. M., Wolff H. A., Neumann K. (2013). Ionizing radiation regulates cardiac Ca handling via increased ROS and activated CaMKII. *Basic Research in Cardiology*.

[63] Ramirez-Correa G. A., Cortassa S., Stanley B., Gao W. D., Murphy A. M. (2010). Calcium sensitivity, force frequency relationship and cardiac troponin I: critical role of PKA and PKC phosphorylation sites. *Journal of Molecular and Cellular Cardiology*.

[64] Kim M., Otsubo R., Morikawa H. (2014). Bacterial effectors and their functions in the ubiquitin-proteasome system: insight from the modes of substrate recognition. *Cell*.

[65] Obin M., Shang F., Gong X., Handelman G., Blumberg J., Taylor A. (1998). Redox regulation of ubiquitin-conjugating enzymes: mechanistic insights using the thiol-specific oxidant diamide. *The FASEB Journal*.

[66] Kumar A., Wu H., Collier-Hyams L. S. (2007). Commensal bacteria modulate cullin-dependent signaling via generation of reactive oxygen species. *The EMBO Journal*.

[67] Reinheckel T., Ullrich O., Sitte N., Grune T. (2000). Differential impairment of 20S and 26S proteasome activities in human hematopoietic K562 cells during oxidative stress. *Archives of Biochemistry and Biophysics*.

[68] Liu J.-Z., Hu Y.-L., Feng Y. (2020). BDH2 triggers ROS-induced cell death and autophagy by promoting Nrf2 ubiquitination in gastric cancer. *Journal of Experimental & Clinical Cancer Research*.

[69] Su X., Shen Z., Yang Q. (2019). Vitamin C kills thyroid cancer cells through ROS-dependent inhibition of MAPK/ERK and PI3K/AKT pathways via distinct mechanisms. *Theranostics*.

[70] Sajadimajd S., Khazaei M. (2018). Oxidative stress and cancer: the role of Nrf2. *Current Cancer Drug Targets*.

[71] Jiang K., Zhang C., Yu B. (2017). Autophagic degradation of FOXO3a represses the expression of PUMA to block cell apoptosis in cisplatin-resistant osteosarcoma cells. *American Journal of Cancer Research*.

[72] Hagenbuchner J., Ausserlechner M. J. (2013). Mitochondria and FOXO3: breath or die. *Frontiers in Physiology*.

[73] van Ooijen H., Hornsveld M., Veen C. D.-d. (2018). Assessment of functional phosphatidylinositol 3-kinase pathway activity in cancer tissue using forkhead box-O target gene expression in a knowledge-based computational model. *The American Journal of Pathology*.

[74] Patra K. C., Hay N. (2014). The pentose phosphate pathway and cancer. *Trends in Biochemical Sciences*.

[75] Ren W., Qiang C., Gao L. (2014). Circulating microRNA-21 (MIR-21) and phosphatase and tensin homolog (PTEN) are promising novel biomarkers for detection of oral squamous cell carcinoma. *Biomarkers*.

[76] Sonenberg N., Hinnebusch A. G. (2009). Regulation of translation initiation in eukaryotes: mechanisms and biological targets. *Cell*.

[77] Ruggero D. (2013). Translational control in cancer etiology. *Cold Spring Harbor Perspectives in Biology*.

[78] Silvera D., Formenti S. C., Schneider R. J. (2010). Translational control in cancer. *Nature Reviews Cancer*.

[79] Bhat M., Robichaud N., Hulea L., Sonenberg N., Pelletier J., Topisirovic I. (2015). Targeting the translation machinery in cancer. *Nature Reviews Drug Discovery*.

[80] Silvera D., Arju R., Darvishian F. (2009). Essential role for eIF4GI overexpression in the pathogenesis of inflammatory breast cancer. *Nature Cell Biology*.

[81] Liang S., Zhou Y., Chen Y., Ke G., Wen H., Wu X. (2014). Decreased expression of EIF4A1 after preoperative brachytherapy predicts better tumor-specific survival in cervical cancer. *International Journal of Gynecologic Cancer*.

[82] Ueda T., Watanabe-Fukunaga R., Fukuyama H., Nagata S., Fukunaga R. (2004). Mnk2 and Mnk1 are essential for constitutive and inducible phosphorylation of eukaryotic initiation factor 4E but not for cell growth or development. *Molecular and Cellular Biology*.

[83] Robichaud N., Sonenberg N. (2014). eIF4E and its binding proteins. *Translation and Its Regulation in Cancer Biology and Medicine*.

[84] Ueda T., Sasaki M., Elia A. J. (2010). Combined deficiency for MAP kinase-interacting kinase 1 and 2 (Mnk1 and Mnk2) delays tumor development. *Proceedings of the National Academy of Sciences*.

[85] Demosthenous C., Han J. J., Stenson M. J. (2015). Translation initiation complex eIF4F is a therapeutic target for dual mTOR kinase inhibitors in non-Hodgkin lymphoma. *Oncotarget*.

[86] Hsieh A. C., Liu Y., Edlind M. P. (2012). The translational landscape of mTOR signalling steers cancer initiation and metastasis. *Nature*.

[87] Mamane Y., Petroulakis E., Rong L., Yoshida K., Ler L. W., Sonenberg N. (2004). eIF4E - from translation to transformation. *Oncogene*.

[88] Avdulov S., Li S., Van Michalek D. B. (2004). Activation of translation complex eIF4F is essential for the genesis and maintenance of the malignant phenotype in human mammary epithelial cells. *Cancer Cell*.

[89] Ruggero D., Montanaro L., Ma L. (2004). The translation factor eIF-4E promotes tumor formation and cooperates with c-Myc in lymphomagenesis. *Nature Medicine*.

[90] Gandin V., Miluzio A., Barbieri A. M. (2008). Eukaryotic initiation factor 6 is rate-limiting in translation, growth and transformation. *Nature*.

[91] Zhang Y., Yu J. J., Tian Y. (2015). eIF3a improve cisplatin sensitivity in ovarian cancer by regulating XPC and p27Kip1 translation. *Oncotarget*.

[92] Fan Y., Guo Y. (2015). Knockdown of eIF3D inhibits breast cancer cell proliferation and invasion through suppressing the Wnt/*β*-catenin signaling pathway. *International Journal of Clinical and Experimental Pathology*.

[93] Gao Y., Teng J., Hong Y. (2015). The oncogenic role of EIF3D is associated with increased cell cycle progression and motility in prostate cancer. *Medical Oncology*.

[94] Lee J. Y., Kim H. J., Rho S. B., Lee S. H. (2016). eIF3f reduces tumor growth by directly interrupting clusterin with anti-apoptotic property in cancer cells. *Oncotarget*.

[95] Qiao Q., Sun C., Han C., Han N., Zhang M., Li G. (2017). Endoplasmic reticulum stress pathway PERK-eIF2*α* confers radioresistance in oropharyngeal carcinoma by activating NF-*κ*B. *Cancer Science*.

[96] Koromilas A. E., Mounir Z. (2013). Control of oncogenesis by eIF2*α* phosphorylation: implications in PTEN and PI3K–Akt signaling and tumor treatment. *Future Oncology*.

[97] Gantenbein N., Bernhart E., Anders I. (2018). Influence of eukaryotic translation initiation factor 6 on non–small cell lung cancer development and progression. *European Journal of Cancer*.

[98] Ricciardi S., Miluzio A., Brina D. (2015). Eukaryotic translation initiation factor 6 is a novel regulator of reactive oxygen species-dependent megakaryocyte maturation. *Journal of Thrombosis and Haemostasis*.

[99] Liu R. R., Lv Y. S., Tang Y. X. (2016). Eukaryotic translation initiation factor 5A2 regulates the migration and invasion of hepatocellular carcinoma cells via pathways involving reactive oxygen species. *Oncotarget*.

[100] Chaurasia M., Gupta S., Das A., Dwarakanath B. S., Simonsen A., Sharma K. (2019). Radiation induces EIF2AK3/PERK and ERN1/IRE1 mediated pro-survival autophagy. *Autophagy*.

[101] Miyasaka H., Endo S., Shimizu H. (2010). Eukaryotic translation initiation factor 1 (eIF1), the inspector of good AUG context for translation initiation, has an extremely bad AUG context. *Journal of Bioscience and Bioengineering*.

[102] Sha Z., Brill L. M., Cabrera R. (2009). The eIF3 interactome reveals the translasome, a supercomplex linking protein synthesis and degradation machineries. *Molecular Cell*.

[103] Walsh D., Perez C., Notary J., Mohr I. (2005). Regulation of the translation initiation factor eIF4F by multiple mechanisms in human cytomegalovirus-infected cells. *Journal of Virology*.

[104] Zhou S. L., Zhou Z. J., Hu Z. Q. (2016). Tumor-associated neutrophils recruit macrophages and T-regulatory cells to promote progression of hepatocellular carcinoma and resistance to sorafenib. *Gastroenterology*.

[105] Kunstman J. W., Juhlin C. C., Goh G. (2015). Characterization of the mutational landscape of anaplastic thyroid cancer via whole-exome sequencing. *Human Molecular Genetics*.

[106] Lobo M. V., Martín M. E., Pérez M. I. (2000). Levels, phosphorylation status and cellular localization of translational factor eIF2 in gastrointestinal carcinomas. *The Histochemical Journal*.

[107] He Y., Yu H., Rozeboom L. (2017). LAG-3 protein expression in non-small cell lung cancer and its relationship with PD-1/PD-L1 and tumor-infiltrating lymphocytes. *Journal of thoracic oncology : official publication of the International Association for the Study of Lung Cancer*.

[108] Tejada S., Lobo M. V., García-Villanueva M. (2009). Eukaryotic initiation factors (eIF) 2alpha and 4E expression, localization, and phosphorylation in brain tumors. *Journal of Histochemistry & Cytochemistry*.

[109] Wang W., Zhao W., Wang H. (2012). Poorer prognosis and higher prevalence of BRAF V600E mutation in synchronous bilateral papillary thyroid carcinoma. *Annals of Surgical Oncology*.

[110] Haybaeck J., O'Connor T, Spilka R. (2010). Overexpression of p150, a part of the large subunit of the eukaryotic translation initiation factor 3, in colon cancer. *Anticancer Research*.

[111] Xu B., Chen L., Li J. (2016). Prognostic value of tumor infiltrating NK cells and macrophages in stage II+ III esophageal cancer patients. *Oncotarget*.

[112] Hao J., Liang C., Jiao B. (2015). Eukaryotic translation initiation factor 3, subunit C is overexpressed and promotes cell proliferation in human glioma U-87 MG cells. *Oncology Letters*.

[113] He J., Wang X., Cai J., Wang W., Qin X. (2017). High expression of eIF3d is associated with poor prognosis in patients with gastric cancer. *Cancer Management and Research*.

[114] Zhu Q., Qiao G. L., Zeng X. C. (2016). Elevated expression of eukaryotic translation initiation factor 3H is associated with proliferation, invasion and tumorigenicity in human hepatocellular carcinoma. *Oncotarget*.

[115] Ahlemann M., Zeidler R., Lang S., Mack B., Münz M., Gires O. (2006). Carcinoma-associated eIF3i overexpression facilitates mTOR-dependent growth transformation. *Molecular Carcinogenesis*.

[116] Goh S.-H., Hong S.-H., Hong S.-H. (2011). eIF3m expression influences the regulation of tumorigenesis-related genes in human colon cancer. *Oncogene*.

[117] Doldan A., Chandramouli A., Shanas R. (2008). Loss of the eukaryotic initiation factor 3f in pancreatic cancer. *Molecular Carcinogenesis*.

[118] Korets S. B., Czok S., Blank S. V., Curtin J. P., Schneider R. J. (2011). Targeting the mTOR/4E-BP pathway in endometrial cancer. *Clinical Cancer Research*.

[119] Haydon M. S., Googe J. D., Sorrells D. S., Ghali G. E., Li B. D. (2000). Progression of eIF4e gene amplification and overexpression in benign and malignant tumors of the head and neck. *Cancer*.

[120] Crew J. P., Fuggle S., Bicknell R., Cranston D. W., De Benedetti A., Harris A. L. (2000). Eukaryotic initiation factor-4E in superficial and muscle invasive bladder cancer and its correlation with vascular endothelial growth factor expression and tumour progression. *British Journal of Cancer*.

[121] Lee J. W., Choi J. J., Lee K. M. (2005). eIF-4E expression is associated with histopathologic grades in cervical neoplasia. *Human Pathology*.

[122] Graff J. R., Konicek B. W., Lynch R. L. (2009). eIF4E activation is commonly elevated in advanced human prostate cancers and significantly related to reduced patient survival. *Cancer Research*.

[123] Berkel H. J., Turbat-Herrera E. A., Shi R., de Benedetti A. (2001). Expression of the translation initiation factor eIF4E in the polyp-cancer sequence in the colon. *Cancer Epidemiology and Prevention Biomarkers*.

[124] Wang X. L., Cai H. P., Ge J. H., Su X. F. (2012). Detection of eukaryotic translation initiation factor 4E and its clinical significance in hepatocellular carcinoma. *World journal of gastroenterology: WJG*.

[125] Yang S. X., Hewitt S. M., Steinberg S. M., Liewehr D. J., Swain S. M. (2007). Expression levels of eIF4E, VEGF, and cyclin D1, and correlation of eIF4E with VEGF and cyclin D1 in multi-tumor tissue microarray. *Oncology Reports*.

[126] Hsu H. S., Chen H. W., Kao C. L., Wu M. L., Li A. F. Y., Cheng T. H. (2011). MDM2 is overexpressed and regulated by the eukaryotic translation initiation factor 4E (eIF4E) in human squamous cell carcinoma of esophagus. *Annals of Surgical Oncology*.

[127] Chen C. N., Hsieh F. J., Cheng Y. M., Lee P. H., Chang K. J. (2004). Expression of eukaryotic initiation factor 4E in gastric adenocarcinoma and its association with clinical outcome. *Journal of Surgical Oncology*.

[128] Tu L., Liu Z., He X. (2010). Over-expression of eukaryotic translation initiation factor 4 gamma 1 correlates with tumor progression and poor prognosis in nasopharyngeal carcinoma. *Molecular Cancer*.

[129] Braunstein S., Karpisheva K., Pola C. (2007). A hypoxia-controlled cap-dependent to cap-independent translation switch in breast cancer. *Molecular Cell*.

[130] Wagner S. D., Willis A. E., Beck D. (2014). eIF4G. *In Translation and Its Regulation in Cancer Biology and Medicine*.

[131] Parsyan A., Sullivan R. J., Meguerditchian A. N., Meterissian S. (2014). Melanoma and Non-Melanoma Skin Cancers. *In Translation and Its Regulation in Cancer Biology and Medicine*.

[132] Nasr Z., Robert F., Porco J. A., Muller W. J., Pelletier J. (2013). eIF4F suppression in breast cancer affects maintenance and progression. *Oncogene*.

[133] Shaoyan X., Juanjuan Y., Yalan T., Ping H., Jianzhong L., Qinian W. (2013). Downregulation of EIF4A2 in non-small-cell lung cancer associates with poor prognosis. *Clinical Lung Cancer*.

[134] Horvilleur E., Sbarrato T., Hill K. (2014). A role for eukaryotic initiation factor 4B overexpression in the pathogenesis of diffuse large B-cell lymphoma. *Leukemia*.

[135] Bai Y., Lu C., Zhang G. (2017). Overexpression of miR-519d in lung adenocarcinoma inhibits cell proliferation and invasion via the association of eIF4H. *Tumour biology : the journal of the International Society for Oncodevelopmental Biology and Medicine*.

[136] Shek F. H., Fatima S., Lee N. P. (2012). Implications of the Use of Eukaryotic Translation Initiation Factor 5A (eIF5A) for Prognosis and Treatment of Hepatocellular Carcinoma. *International journal of hepatology*.

[137] Preukschas M., Hagel C., Schulte A. (2012). Expression of eukaryotic initiation factor 5A and hypusine forming enzymes in glioblastoma patient samples: implications for new targeted therapies. *PLoS One*.

[138] Caraglia M., Park M. H., Wolff E. C., Marra M., Abbruzzese A. (2013). eIF5A isoforms and cancer: two brothers for two functions?. *Amino Acids*.

[139] Chen W., Luo J.-H., Hua W.-F. (2009). Overexpression of EIF-5A2 is an independent predictor of outcome in patients of urothelial carcinoma of the bladder treated with radical cystectomy. *Cancer Epidemiology and Prevention Biomarkers*.

[140] Yang G. F., Xie D., Liu J. H. (2009). Expression and amplification of eIF-5A2 in human epithelial ovarian tumors and overexpression of EIF-5A2 is a new independent predictor of outcome in patients with ovarian carcinoma. *Gynecologic Oncology*.

[141] Bao Y., Lu Y., Wang X. (2015). Eukaryotic translation initiation factor 5A2 (eIF5A2) regulates chemoresistance in colorectal cancer through epithelial mesenchymal transition. *Cancer Cell International*.

[142] Weis F., Giudice E., Churcher M. (2015). Mechanism of eIF6 release from the nascent 60S ribosomal subunit. *Nature Structural & Molecular Biology*.

[143] Flavin R. J., Smyth P. C., Finn S. P. (2008). Altered eIF6 and Dicer expression is associated with clinicopathological features in ovarian serous carcinoma patients. *Modern Pathology*.

